# Dynamic resource allocation in spatial working memory during full and partial report tasks

**DOI:** 10.1167/jov.23.2.10

**Published:** 2023-02-21

**Authors:** Siobhan M. McAteer, Emma Ablott, Anthony McGregor, Daniel T. Smith

**Affiliations:** 1 Department of Psychology, Durham University, Durham, UK

**Keywords:** visuospatial working memory, working memory, serial order, resource allocation

## Abstract

Serial position effects are well-documented in working memory literature. Studies of spatial short-term memory that rely on binary response; full report tasks tend to report stronger primacy than recency effects. In contrast, studies that utilize a continuous response, partial report task report stronger recency than primacy effects (Gorgoraptis, Catalao, Bays, & Husain, 2011; Zokaei, Gorgoraptis, Bahrami, Bays, & Husain, 2011). The current study explored the idea that probing spatial working memory using full and partial continuous response tasks would produce different distributions of visuospatial working memory resources across spatial sequences and, therefore, explain the conflicting results in the literature. Experiment 1 demonstrated that primacy effects were observed when memory was probed with a full report task. Experiment 2 confirmed this finding while controlling eye movements. Critically, Experiment 3 demonstrated that switching from a full to a partial report task abolished the primacy effect and produced a recency effect, consistent with the idea that the distribution of resources in visuospatial working memory depends on the type of recall required. It is argued that the primacy effect in the whole report task arose from the accumulation of noise caused by the execution of multiple spatially directed actions during recall, whereas the recency effect in the partial report task reflects the redistribution of preallocated resources when an anticipated item is not presented. These data show that it is possible to reconcile apparently contradictory findings within the resource theory of spatial working memory and the importance of considering how memory is probed when interpreting behavioral data through the lens of resource theories of spatial working memory.

## Introduction

Visuospatial working memory (VSWM) is the limited capacity store for the temporary maintenance and manipulation of spatial and nonspatial (visual) information (Baddeley, 2011; Baddeley & Hitch, 1974). There is continued debate surrounding the nature of capacity limitations in VSWM (Fallon, Zokaei, & Husain, 2016; Luck & Vogel, 2013; Ma et al., 2014). One influential idea is that VSWM is a flexible and dynamic resource, which is distributed across all task-relevant items (Bays, Catalao, & Husain, 2009). The precision with which information is retained is dependent on the proportion of resource directed to each item. The resource model of VSWM has received considerable behavioral and neuroscientific support (for reviews, Fallon et al., 2016; Ma, Husain, & Bays, 2014). However, the ways in which resources are distributed across visuospatial sequences are not well-understood. To examine the redistribution of resources across a sequence in VSWM, we investigated how memory for spatial locations and the corresponding response errors differ depending on set size and serial position.

Serial position effects have typically been examined in verbal memory tasks, where participants are asked to recall sequences of words. Studies have shown a primacy effect, where there is a sharp monotonic decrease in recall accuracy from the first serial position. There is also a small recency effect, where performance improves for the final item in the sequence (Murdock, 1968; Saint-Aubin & Poirier, 2000). These effects in verbal memory have been replicated in visuospatial memory (Guérard & Tremblay, 2008; Jones, Farrand, Stuart, & Morris, 1995; Martín, Tapper, González, Leclerc, & Niechwiej-Szwedo, 2017; Smyth & Scholey, 1996). Specifically, in spatial memory, Guérard and Tremblay (2008) asked participants to reconstruct a sequence of spatial locations after presentation of seven black dots. Performance was compared with a verbal memory task. The serial position curves observed in both spatial and verbal tasks were similar, exhibiting primacy and small recency effects. Transposition errors were more likely than omission errors, where no item is recalled, and this was found to increase across serial positions in both verbal and spatial tasks (Guérard & Tremblay, 2008). These effects have also been observed when visual-spatial movements (Agam, Bullock, & Sekuler, 2005; Agam, Galperin, Gold, & Sekuler, 2007; Agam, Huang, & Sekuler, 2010) and auditory–spatial locations (Parmentier & Jones, 2000; Tremblay, Parmentier, Guérard, Nicholls, & Jones, 2006) were examined, suggesting a reliable serial position effect across domains.

Studies examining serial position effects in VSWM have typically relied on binary response tasks, especially Corsi blocks task (Milner, 1971). Although this task provides a reliable measurement of spatial working memory (Vandierendonck, Kemps, Fastame, & Szmalec, 2004), the use of a binary response permits limited examination of the representations maintained in VSWM. The pattern of response errors across serial positions and how this might relate to the distribution of VSWM resources therefore remains unclear. The continuous report task (Wilken & Ma, 2004), which requires participants to reproduce a feature along a continuous dimension, permits a more detailed examination of VSWM representations and the sources of recall error (Bays, Catalao, & Husain, 2009; Zokaei, Burnett Heyes, Gorgoraptis, Budhdeo, & Husain, 2015). The continuous report task has been used extensively to investigate the representations of visual (Bays et al., 2009; Zhang & Luck, 2008) and spatial (Pertzov, Dong, Peich, & Husain, 2012; Schneegans & Bays, 2016) features in VSWM. Behavioral studies using this approach to examine serial position effects indicate that the redistribution of VSWM resources across a sequence may not follow the serial position curve observed for quantized response tasks, in contrast with most models of serial order effects (Gorgoraptis, Catalao, Bays, & Husain, 2011; Zokaei, Gorgoraptis, Bahrami, Bays, & Husain, 2011). Specifically, Gorgoraptis et al. (2011) showed that, as the number of to-be-remembered items increased, precision in memory for orientation decreased monotonically. Across a sequence of to-be-remembered items, a strong recency effect was observed: precision was highest, with the lowest probability of misbinding, for the final presented item.

Such a strong recency effect was proposed to reflect dynamic redistribution of VSWM resources toward the most recently presented item. However, it is at odds with previous empirical work examining verbal and spatial working memory (Guérard & Tremblay, 2008) and is inconsistent with the predictions of computational models of serial position effects, which predict small recency effects (Hurlstone, Hitch, & Baddeley, 2014). One potential reason for this difference is that participants exposed to the continuous report paradigms did not necessarily have to rely on spatial representations to solve the task. The task used by Gorgoraptis et al. (2011) could be carried out without relying on spatial location because the test orientation was probed by color, even when spatial location was randomized in their Experiment 3. Their task relied only on memory for visual features rather than memory for visual–spatial conjunctions, which might explain why the serial position curve differed from previous findings in VSWM. The current study aimed to investigate the distribution of VSWM resources across sequences of spatial locations by examining the pattern of response errors across serial positions in a spatial continuous report task. This strategy permits the examination of whether performance in a spatial continuous report task, where visual and spatial features must be remembered, mirrors that of verbal tasks (Guérard & Tremblay, 2008), spatial tasks (Martín et al., 2017), or visual tasks (Gorgoraptis et al., 2011).

## Experiment 1

### Methods

#### Participants

An a priori power analysis was carried out in G*Power v3.1 (Faul, Erdfelder, Lang, & Buchner, 2007). We based this on Gorgoraptis et al. (2011), who reported a large effect of serial order on precision ($\eta_{p}^{2}$ = .27). We carried out a power analysis to detect a main effect of serial position using within-subjects analysis of variance with a factor of serial position with up to four levels, 90% power, and an alpha value of 0.05. The analysis indicated at least ten participants would be required for a set size of two, eight participants at set size three, and seven participants at set size four. We recruited eight students from Durham University (*M_age_* = 23.75 years, *SD_age_* = 4.13, 5 females, 3 males, 7 right handed, 1 left handed). Participants were compensated at a rate of £8/h for their time. This study received ethical approval from the Department of Psychology Research Ethics Committee (reference: PSYCH-2019-10-28T15:23:58-lckd86).

#### Design

We used a mixed design with two independent variables. Our between-subjects independent variable was presentation mode (2 levels: sequential and simultaneous), and our within-subjects independent variable was set size (4 levels: 1, 2, 3, and 4 items). Serial position (≤4 levels) was an additional within-subjects independent variable for the sequential presentation condition. The dependent variables were imprecision, and the probabilities of reporting the target location(s), nontarget location(s), and guessing.

Participants were randomly assigned to one of the two presentation conditions. They first completed a block of eight practice trials, two of each set size. Practice trials were the same as the experimental trials, with the exception that participants were shown the correct locations as well as their own responses on screen. Once the practice trials were completed, participants completed 320 experimental trials, 80 of each set size, randomized across 16 blocks. Participants were free to take a self-paced break between blocks.

#### Stimuli and apparatus

The task was programmed using Matlab R2019a, using the psychophysics toolbox (Kleiner, Brainard, & Pelli, 2007). The stimuli consisted of arrays comprising one, two, three, or four colored dots (radius = 0.5 visual angle) and a fixation cross positioned at the center of the screen (height of fixation cross = 1 visual angle). The colors of each dot were chosen without repetition from a bank of seven distinct colors: red, orange, green, cyan, blue, magenta, and purple. The locations of the dots were chosen randomly within the annular region 5° to 10° of visual angle around central fixation, with at least 1.5 visual angle separating each dot. The visual mask comprised 800 colored dots, like those presented at encoding, filling the annular space 5° to 10° of visual angle around central fixation. Participants were asked to respond as accurately and as quickly as possible. Participants were unaware that the area of stimulus presentation was constrained and were free to respond with any location on screen. Participants’ gaze was monitored using a tower-mounted EyeLink 1000 eye tracker (SR Research). Stimuli were presented on a 20-inch CRT screen with a refresh rate of 85Hz. Participants sat 60 cm from the computer screen, with the center of the screen at eye level.

#### Procedure

**Figure 1.**
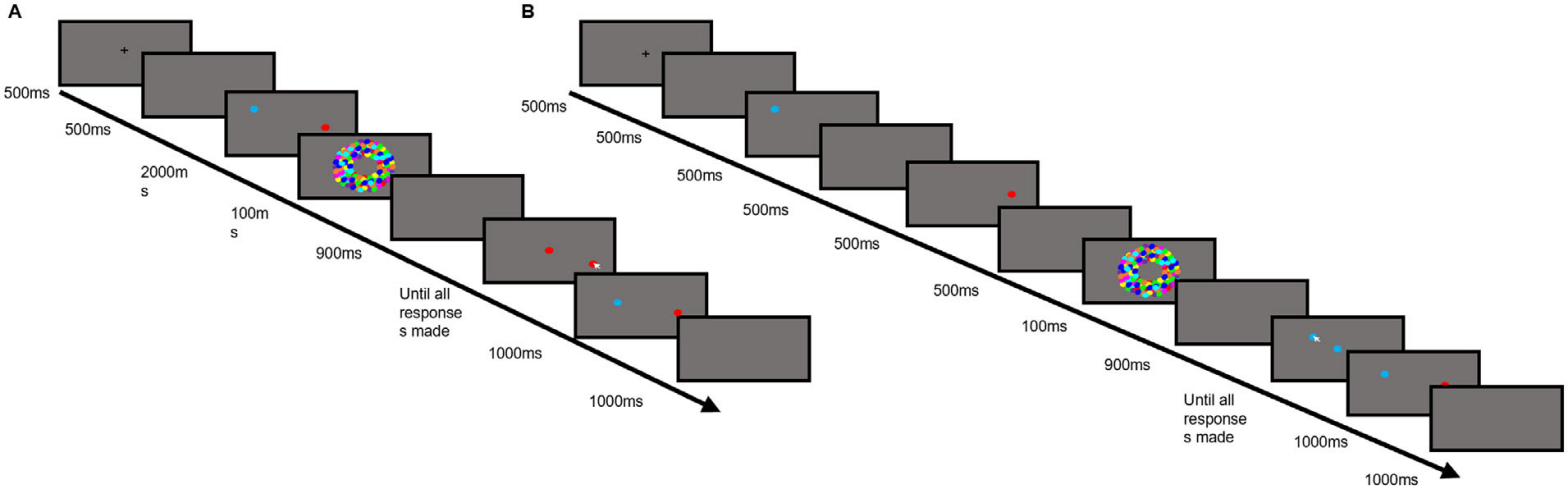
An example trial for simultaneous (A) and sequential (B) conditions.

Trials began with presentation of a fixation cross at the center of the screen for 500 ms, followed by a blank screen for 500 ms. The stimulus array, comprising one, two, three, or four colored dots, was then presented. In the simultaneous presentation condition, the array was shown for 2,000 ms. In the sequential presentation condition, each dot was shown for 500 ms, with a 500-ms interstimulus delay. After presentation of the array, the visual mask was presented for 100 ms, followed by a 900-ms blank screen. Participants were then asked to respond with the locations of each dot presented on that trial, indexed by the colored dot being shown at the center of the screen and changing color when the mouse was clicked. In sequential conditions, the order of responding was the same as the order of presentation. There was no time limit for responding. Responses were shown as an array on screen for 1,000 ms, after which a 1,000-ms blank screen was shown, before the beginning of the next trial. An example trial for each condition is shown in Figure 1.

#### Statistical analyses

Mixture modelling (Bays et al., 2009) was carried out using MemToolbox2D (Grogan et al., 2020). This mixture model (Bays et al., 2009) assumes that there are three sources of recall error: Gaussian variability in the response (imprecision), the height of which indicates the probability of reporting the target location; the probability of guessing, which is drawn from a uniform distribution; and the probability of responding with a nontarget (misbinding), which is drawn from a Gaussian centered on one of the nonprobed items. Maximum likelihood estimates were obtained for these sources of recall error in each condition. The estimate of guessing was corrected by assuming that responses were sampled from the annulus within which items could appear. Comparison of the corrected Akaike information criterion (AIC) values for the model with and without this response sampling showed that the model that assumes response sampling provided a better fit in all experiments for all participants (Δ*AICc*_*Experiment* 1_  = 20.19; Δ*AICc*_*Experiment* 2_  = 38.95; Δ*AICc*_*Experiment* 3_  = 30.69.

### Results

An analysis of the effect of presentation mode is presented in Supplementary Materials S1. Briefly, there were no significant effects of presentation mode or interactions between presentation mode and set size. For the current analysis, we report only the data from the sequential presentation mode. We included all datasets in this condition. Examination of the eye tracking data revealed that, on average, approximately 20 saccades were made on each trial (*M* = 19.87, *SD* = 8.33, minimum = 1, maximum = 51).

Owing to the small sample size, we ran linear mixed effects model in R version 4.2.1 (R Core Team, 2019) using the lmerTest package (Kuznetsova, Brockhoff, & Christensen, 2017), which applies Satterthwaite's method to estimate degrees of freedom and *p* values for the overall effect of serial position. The model was run on each set size to examine the effect of serial position after controlling for the random effect of participant. Serial position was included as a fixed effect and we included participant ID as a random effect.^1^ Bonferroni–Holm corrected post hoc contrasts of the estimated marginal means were carried out to examine any significant effects, using the emmeans package (Lenth, 2022).

**Figure 2.**
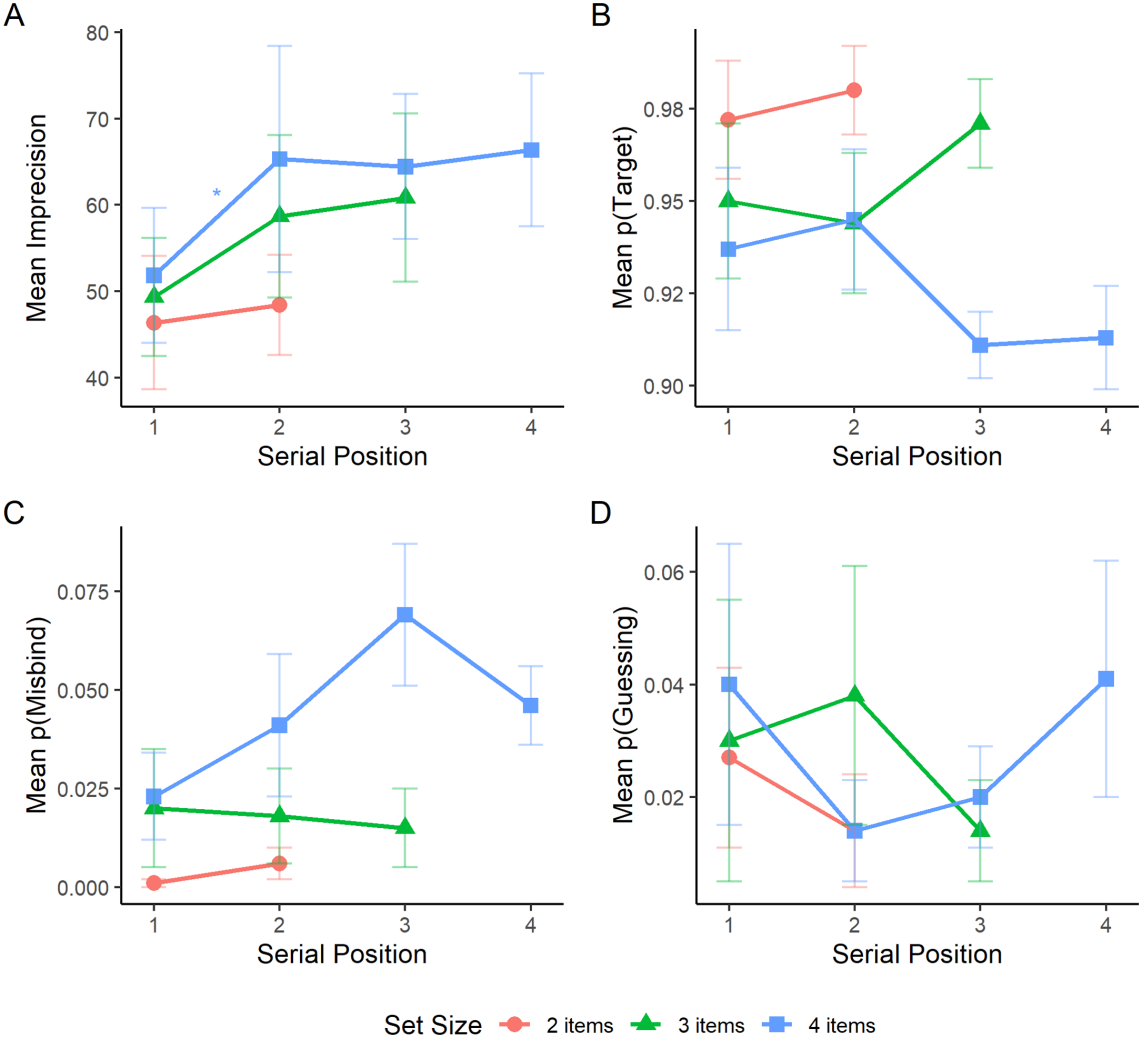
Mean imprecision (A), probability of reporting the target location (B), probability of misbinding (C), and probability of guessing (D) for each set size in the sequential presentation condition as a function of serial position. Error bars represent the standard error of the mean.

For imprecision (Figure 2A), a significant effect of serial position was observed at set size four, *F*(3, 9) = 4.96, *p* = 0.027. The effect of serial position was not significant at set size two, *F*(1, 3) = 0.37, *p* = 0.587, or set size three, *F*(2, 6) = 4.33, *p* = 0.069. Post hoc pairwise comparisons between serial positions revealed a significant difference between the first and second item at set size four (*p* = 0.037). No other comparisons were significant (*p* ≥ 1.000). For the probability of reporting the target (Figure 2B), no significant effects of serial position were observed at set sizes two, *F*(1, 3) = 0.55, *p* = 0.513, three, *F*(2, 6) = 1.12, *p* = 0.387, and four, *F*(3, 9) = 1.22, *p* = 0.357. For the probability of misbinding (Figure 2C), a significant effect of serial position was observed at set size four, *F*(3, 9) = 4.67, *p* = 0.031. The effect of serial position was not significant at set sizes two, *F*(1, 3) = 2.73, *p* = 0.197, and three, *F*(2, 9) = 0.06, *p* = 0.946. Post hoc pairwise comparisons between serial positions revealed no significant differences between items at set size four (*p* ≥ 0.145). For the probability of guessing (Figure 2D), the effect of serial position was not significant at any set size; set size two, *F*(1, 3) = 0.96, *p* = 0.400; set size three, *F*(2, 9) = 0.35, *p* = 0.715; set size four, *F*(3, 12) = 0.62, *p* = 0.617.

### Discussion

This experiment aimed to examine the distribution of VSWM resources across sequences of spatial locations. We observed a weak primacy effect; the first presented item was remembered more precisely than items presented later in the sequence, although these effects did not reach significance. There were no effects of serial position or set size on the probability of guessing, indicating that all items were encoded into memory but with increasing noise as the sequence length increased. This pattern of results is broadly consistent with prior work that reports primacy effects in tasks that measure spatial working memory (Martín et al., 2017). However, these results are inconsistent with previous work using visual continuous report tasks (Gorgoraptis et al., 2011; Zokaei et al., 2011).

There are key differences in our task and previous work (Gorgoraptis et al., 2011; Zokaei et al., 2011) that might account for the differences in the serial position effects observed. First, central fixation was not enforced in our experiment, but was enforced in Gorgoraptis et al. (2011). Although participants tend to make few on-item fixations when encoding under free viewing conditions (Lange & Engbert, 2013; Patt et al., 2014; Souza, Czoschke, & Lange, 2020), Saint-Aubin, Tremblay, and Jalbert (2007) have shown that the number and duration of fixations on task-relevant items is positively correlated with recall performance. Similarly, Martín et al. (2017) observed that target-directed saccades improved recall accuracy for small set sizes. It may be that allowing participants to move their eyes freely throughout encoding and maintenance improved recall for the first item, but noise accumulated in the sequence of eye movements throughout encoding and maintenance, resulting in increased imprecision for these items.

The second key difference is that we used a whole report task, whereas Gorgoraptis et al. (2011) and Zokaei et al. (2011) used a single probe task. It is well-documented that the nature of the recall task affects recall performance (Sperling, 1960). Asking participants to recall one item from a sequence may result in VSWM resources being distributed across a sequence differently from when the whole sequence must be reported.

We carried out two further experiments to examine whether these differences underlie the primacy effect observed in the current experiment.

## Experiment 2

We carried out a second experiment to examine whether the difference in viewing conditions between previous work (Gorgoraptis et al., 2011; Zokaei et al., 2011) and Experiment 1 might underlie the differences in the serial order curves observed.

### Methods

#### Participants

We recruited 14 students from Durham University (*M* = 29.43 years, *SD* = 9.18, 9 females, 4 males, 1 nonbinary, 12 right handed). Undergraduate students enrolled on Psychology courses at Durham University (*n* = 3) were credited with participant pool time in exchange for their participation. Other participants (*n* = 11) were compensated at a rate of £8/h for their time. This study received ethical approval from the Department of Psychology Research Ethics Committee (reference: PSYCH-2019-10-28T15:23:58-lckd86).

#### Design

The design was the same as Experiment 1.

#### Stimuli and apparatus

The stimuli and apparatus matched those of Experiment 1.

#### Procedure

The procedure followed Experiment 1, with the exception that participants were asked to maintain central fixation throughout each trial.

### Results

Trials in which average saccade amplitude exceeded 2 VA during encoding and maintenance were excluded from analysis. This process led to the exclusion of four datasets and the exclusion of 16.62% of trials from the remaining 10 datasets. An analysis of the effect of presentation mode is presented in Supplementary Materials S2 and S3. Briefly, there were no significant effects of presentation mode or interactions between presentation mode and set size, and free viewing improved recall precision of single-item displays. The linear mixed effects model matched the serial position analysis in Experiment 1, which included serial position as a fixed effect and participant ID as a random effect.

**Figure 3.**
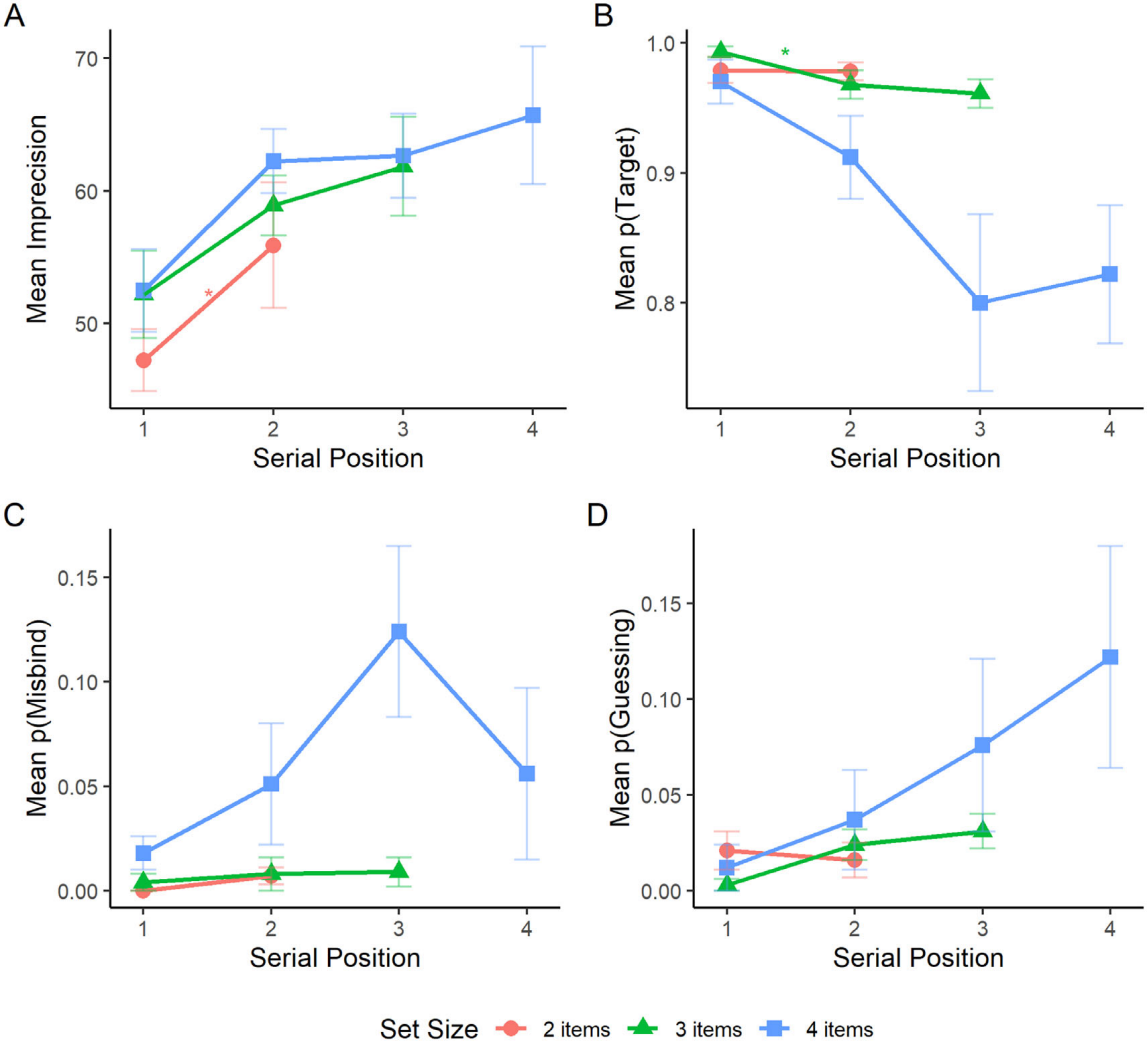
Mean imprecision (A), probability of reporting the target location (B), probability of misbinding (C), and probability of guessing (D) for each set size in the sequential presentation condition as a function of serial position. Error bars represent the standard error of the mean.

For imprecision (Figure 3A), a significant effect of serial position was found at set size two, *F*(1, 4) = 9.01, *p* = 0.040, and set size four, *F*(3, 12) = 4.32, *p* = 0.028. The effect of serial position was not significant at set size three. *F*(2, 8) = 3.41, *p* = 0.085. Post hoc pairwise comparisons using Bonferroni–Holm correction between serial positions revealed no significant differences between items at set size four (*p* ≥ 0.084).

For the probability of reporting the target location (Figure 3B), a significant effect of serial position was observed at set sizes three, *F*(2, 8) = 7.9, *p* = 0.013, and four, *F*(3, 12) = 5.89, *p* = 0.010. The effect of serial position was not significant at set size two, *F*(1, 4) = 0.02, *p* = 0.884. Post hoc pairwise comparisons using Bonferroni–Holm correction between serial positions revealed a significant difference between the first and second presented item at set size three (*p* = 0.036). No other differences were significant at set size three (*p* ≥ 0.435) or set size four (*p* ≥ 0.095).

For the probability of misbinding (Figure 3C), a significant main effect of serial position was found at set size four, *F*(3, 12) = 4.84, *p* = 0.020. There was no significant effect of serial position at set size two, *F*(1, 4) = 2.58, *p* = 0.184, or set size three. *F*(2, 8) = 1.27, *p* = 0.331. Post hoc pairwise comparisons using Bonferroni–Holm correction between serial positions revealed no significant differences at set size four (*p* ≥ 0.075).

For the probability of guessing (Figure 3D), the main effect of serial position was significant at set size three, *F*(2, 8) = 6.85, *p* = 0.018. The effect of serial position was not significant at set sizes two, *F*(1, 4) = 0.33, *p* = 0.599, or four, *F*(3, 12) = 2.3, *p* = 0.129. Post hoc pairwise comparisons using Bonferroni-Holm correction between serial positions revealed no significant differences between items at set size three (*p* ≥ 0.053).

#### Comparison between free (Experiment 1) and fixed (Experiment 2) viewing

**Figure 4.**
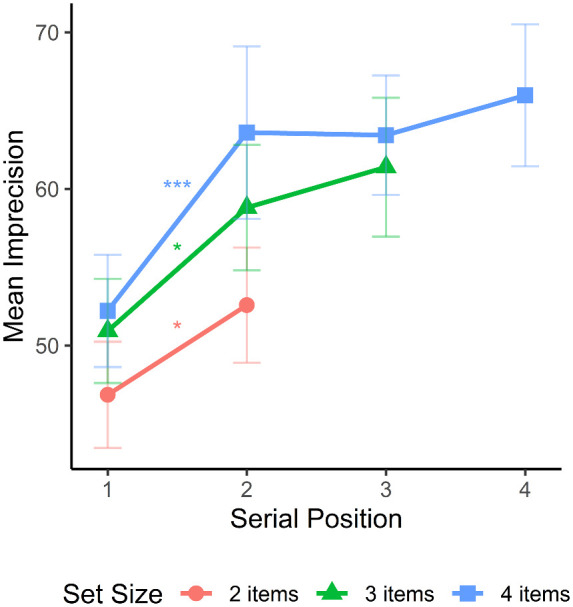
Mean imprecision for each serial position as a function of set size in the sequential presentation conditions collapsed across Experiments 1 and 2. Error bars represent the standard error of the mean.

We examined whether the instruction to maintain central fixation affected imprecision in VSWM using linear mixed effects model (Figure 4). Set size, presentation mode, and viewing condition were included as fixed effects, and participant ID was included as a random effect.^2^ There was no main effect of viewing condition on imprecision at any set size (*p* ≥ 0.573). Serial position and viewing condition did not interact at any set size (*p* ≥ 0.178). The effect of serial position was significant at set size two, *F*(1, 7) = 5.88, *p* = 0.046, set size three, *F*(2, 14) = 7.67, *p* = 0.006, and set size four, *F*(3, 21) = 9.17, *p* < 0.001. The difference between the first and second item was significant at set size three (*p* = 0.026) and set size four (*p* = 0.002). No other differences were significant (*p*_*ss*3_ ≥ 0.383, *p*_*ss*4_ ≥ 0.806).

### Discussion

This experiment examined whether the requirement to maintain central fixation would reverse the recency effect in spatial working memory observed in Experiment 1 to the primacy effect observed in previous studies (Gorgoraptis et al., 2011; Zokaei et al., 2011). Contrary to this idea, we observed a recency effect in Experiment 2. Indeed, when we compared imprecision across Experiments 1 and 2, the primacy effect was stronger in Experiment 2, with the first item of the list being represented more precisely in VSWM than subsequent items. This result seems to rule out the possibility that the discrepancy between our findings and those of Gorgoraptis et al. (2011) and Zokaei et al. (2011) can be explained by participants using different oculomotor strategies. An alternative explanation for the discrepant findings relates to the nature of the task. More specifically, we used a whole report task, whereas previous work (Gorgoraptis et al., 2011; Zokaei et al., 2011) used a partial report task that required participants to recall information about one of the presented items. This difference might have affected how resources were distributed throughout both tasks. In Gorgoraptis et al. (2011) and Zokaei et al. (2011), participants were unaware which item was task relevant. It was argued that resources were distributed toward the most task-relevant item, which was presumed to be the most recent item. Gorgoraptis et al. (2011) subsequently used a cue to explicitly indicate a task-relevant item. Precision was found to increase for the most task-relevant item, regardless of serial position. In contrast, in our task, all items were equally relevant for successful task competition, which is likely to have resulted in resources being distributed differently compared with when only a single probe is used at recall. The possibility was examined in Experiment 3.

It is worth noting that imprecision was lower at set size one in free viewing compared with fixed viewing (see S3). This may be due to the fact that that item was fixated, resulting in a boost in its representation from an additional resource, as outlined in the model proposed by Udale, Tran, Manohar, and Husain (2022).

## Experiment 3

We carried out a third experiment to investigate the possibility that the contrasting effects observed in Experiments 1 and 2 and previous work (Gorgoraptis et al., 2011; Zokaei et al., 2011) are due to differences in the nature of the task.

### Methods

#### Participants

We based the minimum sample size on an a priori power analysis was carried out in G*Power v3.1 (Faul et al., 2007) based on Gorgoraptis et al. (2011), who reported a large effect of serial order for precision ($\eta_{p}^{2}=0.27$). We carried out a power analysis for within-subjects analysis of variance with factor of serial position, with up to four levels, with 90% power and an alpha of 0.05. To detect this main effect at set size three, we required a sample size of at least eight participants, and at least seven were required to detect the same effect at set size four. We recruited 10 students from Durham University (*M* = 19.4 years, *SD* = 0.97 years, 8 females, 1 male, 1 other/prefer not to say, 10 right handed). All participants reported having normal or corrected-to-normal vision. Participants were compensated £10 for their time. This study received ethical approval from the Department of Psychology Research Ethics Committee (reference: PSYCH-2019-10-28T15:23:58-lckd86).

#### Design

Design was the same as Experiment 2, with the exception that only sequential presentation mode was examined. Participants were asked to recall the location of one item from the display at test. Each set size and serial position was tested 80 times over 2 sessions. Each session comprised 20 blocks of 20 trials, resulting in each participant completing a total of 800 trials.

### Stimuli and apparatus

The stimuli and apparatus matched those of Experiments 1 and 2.

#### Procedure

The procedure followed Experiment 2.

### Results

One participant was excluded from the analysis because they did not complete the full experiment. No other data were excluded. The linear mixed effects models matched the serial position analysis in Experiments 1 and 2, which included serial position as a fixed effect and participant ID as a random effect for each set size.

**Figure 5.**
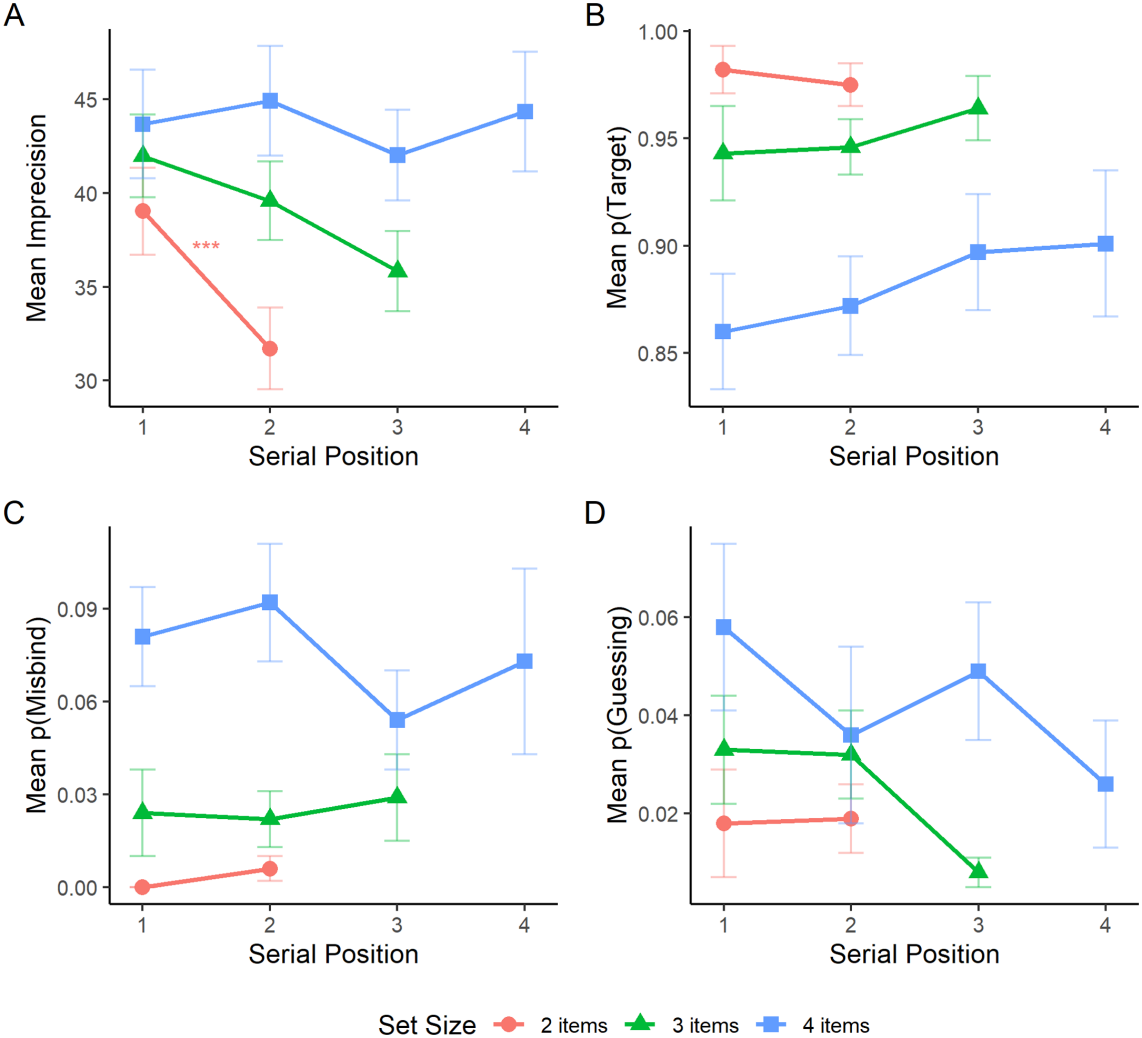
Mean imprecision (A), probability of reporting the target location (B), probability of misbinding (C), and probability of guessing (D) for each set size as a function of serial position. Error bars represent the standard error of the mean.

For imprecision (Figure 5A), a significant effect of serial position was found for set size two, such that imprecision was higher for the first item than the second item in the sequence, *F*(1, 8) = 34.46, *p* < 0.001. The effect of serial position was also significant at set size three, *F*(2, 16) = 5.97, *p* = 0.012, but no pairwise comparisons were significant (*p* ≥ 0.455). The effect of serial position was not significant at set size four, *F*(3, 24) = 0.54, *p* = 0.661. For the probability of reporting the target location (Figure 5B), the effect of serial position was not significant at set size two, *F*(1, 8) = 0.34, *p* = 0.575, three, *F*(2, 16) = 0.74, *p* = 0.492, or four, *F*(3, 24) = 1.21, *p* = 0.328. Similarly, for the probability of misbinding (Figure 5C), the effect of serial position was not significant at set size two, *F*(1, 8) = 2.27, *p* = 0.170, three *F*(2, 16) = 0.13, *p* = 0.876, or four, *F*(3, 24) = 0.9, *p* = 0.453. Finally, for the probability of guessing (Figure 5D), the effect of serial position was not significant at set size two, *F*(1, 8) = 0.01, *p* = 0.917, three, *F*(2, 16) = 3.38, *p* = 0.060, or four, *F*(3, 24) = 1.41, *p* = 0.264.

#### Comparison between the whole report (Experiments 1 and 2) and a single probe (Experiment 3)

We also compared imprecision between our whole report tasks (Experiments 1 and 2) and our single probe task (Experiment 3) to examine whether and how the serial position effect was affected by changing the nature of the recall task. The linear mixed effects model was carried out at each set size and included fixed effects of serial position, and recall task, as well as the interactions between recall task and serial position. We included participant ID as a random effect.^3^

We found a significant main effect of recall task at all set sizes, where imprecision in the whole report task was significantly greater than in the single probe task at each set size; set size two, *F*(1, 16) = 13.05, *p* = 0.002; set size three, *F*(1, 16) = 18.92, *p* < 0.001; and set size four, *F*(1, 16) = 13.55, *p* = 0.002.

**Figure 6.**
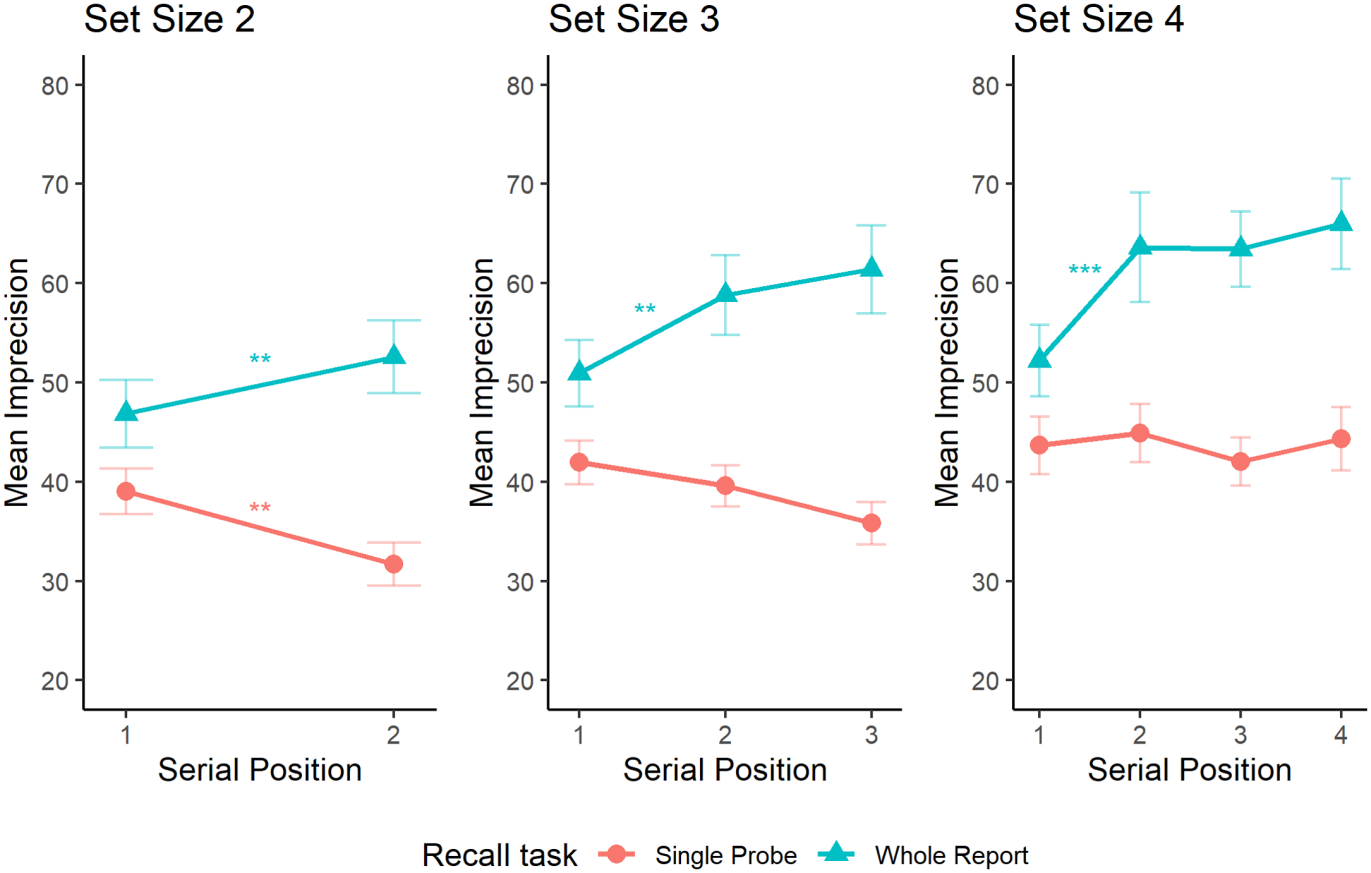
Mean imprecision for whole report (sequential presentation mode only) and single probe tasks as a function of serial position at each set size. Error bars represent the standard error of the mean.

A significant interaction between recall task and serial position was also observed at every set size; set size two, *F*(1, 16) = 23.91, *p* < 0.001; set size three: *F*(2, 32) = 13.79, *p* < 0.001; and set size four, *F*(3, 48) = 5.73, *p* = 0.002. To examine what was driving these interactions, we analyzed the serial position effects for single probe and whole report tasks at each set size (Figure 6). There were significant serial position effects on whole report tasks at all set sizes; set size two, *F*(1, 16) = 9.2, *p* = 0.008; set size three, *F*(2, 32) = 11.68, *p* < 0.001; and set size four, *F*(3, 48) = 11.5, *p* < 0.001. There was a significant serial position effect on the single probe task at set sizes two, *F*(1, 16) = 15.07, *p* = 0.001, and three, *F*(2, 32) = 3.76, *p* = 0.034. The serial position effect was not significant at set size four on the single probe task, *F*(3, 48) = 0.47, *p* = 0.705.

We then compared the serial position effects in each recall task at each set size using Holm–Bonferroni corrected pairwise comparisons. At set size two, there was a primacy effect on the whole report task, where the first item was remembered with greater precision than the second item (*p* = 0.008). This pattern was reversed in the single probe task (*p* = 0.001). At set size three, the first item was remembered more precisely than the second item on the whole report task (*p* = 0.003). No other comparisons were significant for either task (*p* ≥ 0.212). The same pattern was observed at set size four, with the first item being remembered more precisely than the second item on the whole report task (*p* < 0.001). No other comparisons were significant for either task (*p* ≥ 0.656).

### Discussion

This experiment examined the effect of moving from a whole report to a partial report task on the distribution of memory resources. The key finding was of a significant interaction between serial position and report type, such that imprecision significantly decreased with serial position in the partial report task (a recency effect) but significantly increased with serial position in the whole report task (a primacy effect). This recency effect was strongest at set size two, where the first presented item was recalled more precisely than the second presented item. The presence of a recency effect in this experiment suggests that the difference in serial position effects found in Experiments 1 and 2 and those found by Gorgoraptis et al. (2011) and Zokaei et al. (2011) are a consequence of differences in the task used. The fact that changing the task from a whole report to a single report produces a very different pattern of data is consistent with the idea that the difference in the nature of the recall in the task (i.e., whether the task is whole report or single probe report) plays an important role in the distribution of resources.

## General discussion

The current study explored how VSWM resources are distributed across sequences by examining the sources of recall error for sequences of spatial locations. When participants were asked to report all locations presented in a sequence, a primacy effect was observed for imprecision and misbinding, regardless of whether participants were free to move their eyes (Experiment 1) or maintained fixation (Experiment 2). The whole report task also required participants to make a sequence of goal-directed actions, as they were required to move the mouse to the correct location of item *N* while simultaneously maintaining the locations of items *N +* 1, *N +* 2, and so on. Goal-directed actions are known to disrupt VSWM (Lawrence, Myerson, Oonk, & Abrams, 2001; McAteer, McGregor, & Smith, 2023; Pearson & Sahraie, 2003; Smyth & Scholey, 1994) and increase imprecision as each action requires spatial updating that introduces noise into stored spatial representations (McAteer et al., 2023; Peterson, Kelly, & Blumberg, 2019). It, therefore, seems likely that the act of responding itself introduced additional noise into the representations of yet-to-be-reported items, resulting in a cumulative increase in precision across serial positions, and a primacy effect in the whole report task. Consistent with this explanation, when four items were presented but only a single response was required in Experiment 3, the serial position effect was abolished (see Figure 6).

When a single probe task was used (Experiment 3), a recency effect was observed. This recency effect is broadly consistent with previous work using a single probe continuous report task in visual working memory (Gorgoraptis et al., 2011; Zokaei et al., 2011). One potential explanation for the existence of the recency effect is the dual-resource model, which proposes that encoding occurs via drawing on one resource pool to attend to an item before drawing on a second resource pool to retain the item after execution of a saccade toward that item (Udale et al., 2022). The implication of this model is that the final saccade target is represented more precisely in memory than preceding items. The recency effect observed in our Experiment 3 was weaker than recency effects reported previously (Gorgoraptis et al., 2011; Udale et al., 2022; Zokaei et al., 2011). This difference may be due to the fact that we did not enforce any viewing strategy. In previous work, participants were required to maintain central fixation (Gorgoraptis et al., 2011) or were instructed make a series of saccades toward items (Udale et al., 2022). These viewing strategies are not reflective of natural viewing behavior, indicating that the strength of the recency effect might also depend on viewing behavior. Additionally, we found that imprecision was relatively stable across serial positions at set size four, which might indicate that participants learned that a maximum of four items would be presented. This learning might have resulted in resources being preallocated across four potential items before encoding. When fewer than four items were presented, the remaining resource was allocated to the final presented item. This flexibility in resource allocation reflects the dynamic nature of VSWM and might represent an efficient strategy for task completion in single probe tasks in which it is not clear which item will be probed. Future work might examine this hypothesis across a larger number of set sizes, and in conditions where participants are instructed as to how many stimuli will be presented.

We also observed that VSWM precision was significantly reduced on the whole report task compared with the single probe task, consistent with the well-documented effect that performance on partial report tasks is better than on whole report tasks (Sperling, 1960). This effect was present for the first item, so cannot be fully explained by the response-induced noise account outlined above in this article. An alternative explanation is that it reflects the use of different reference frames in whole report and single probe tasks. In the whole report task, participants were required to recreate the full sequence of spatial locations at recall. The first item in the sequence was always recalled relative to central fixation, but subsequent items were recalled relative to the previously reported location. Participants may, therefore, have relied on global or relational information and a spatiotopic reference frame. Conversely, in the single probe task, the sequence was almost irrelevant to task completion because recall of the probe item was always cued by the object color not its place in the sequence, and initiated from the center of the screen. The successful recall of the location in the single probe task, therefore, relied on local information and a retinotopic reference frame. The maintenance of spatial locations is associated with activation in retinotopic spatial maps in visual areas and parietal cortex (Ester, Serences, & Awh, 2009; Jerde, Merriam, Riggall, Hedges, & Curtis, 2012; Pratte & Tong, 2014; Serences, Ester, Vogel, & Awh, 2009) consistent with the view that the default reference frame for representing spatial locations is retinotopic (Golomb & Kanwisher, 2012). Transformation from a retinotopic into a spatiotopic reference frame is noisy, leading to decreased memory performance (Golomb & Kanwisher, 2012; Shafer-Skelton & Golomb, 2018). This noisy conversion explanation aligns with our observation that memory was less precise on the whole report task compared with the single probe task at all serial positions.

The flexibility in the use of retinotopic and spatiotopic reference frames highlights the flexibility in VSWM, where the nature of representation depends on task demands (Serences, 2016), which may include the nature of the recall task. However, it should be noted that our range of set sizes (two to four) was somewhat smaller than those typically used to examine serial order effects (set sizes of one to seven), and it is possible that other or larger differences in the distribution of resources across items might emerge at larger set sizes. Additionally, it is possible that some subtle effects were missed in the analysis owing to the small sample sizes used. However, the overall trend of the data indicates that the distribution of resources depends on the nature of the recall task used, which was the key research question.

To summarize, we found evidence for a primacy effect in VSWM when participants were asked to recall all presented spatial locations in sequence (Experiments 1 and 2). These observations are not consistent with previous work using the continuous report task, which reported large recency effects (Gorgoraptis et al., 2011; Zokaei et al., 2011). However, when a single probe task (Experiment 3) was used, a recency effect was observed, which is more consistent with previous work. We propose that the primacy effect in the whole report task arises from the accumulation of noise caused by the execution of multiple spatially directed actions during recall, whereas the recency effect in the partial report task reflects the redistribution of preallocated resources when an anticipated item is not presented. The fact that subtle differences in the nature of the recall task determines how resources are allocated demonstrates the flexible and dynamic nature of resource allocation in VSWM (Bays et al., 2009; Udale et al., 2022).

## Supplementary Material

Supplement 1

## Footnotes

1 For all set sizes, the formula for the model was as follows for imprecision: imprecision ∼ serial position + (1|ID).

2 The formula for the model was as follows: imprecision ∼ set size + serial position + viewing condition + set size*serial position + set size*viewing condition + serial position*viewing condition + set size*serial position*viewing condition + (1|ID).

3 The formula for the model was as follows: imprecision ∼ serial position + recall task + serial position*recall task + (1|ID),
