# Supplementary material for: Dynamic resource allocation in spatial working memory during full and partial report tasks
Source: J Vis. 2023 Feb 21;23(2):10. doi: 10.1167/jov.23.2.10 (PMC9946046; doi:10.1167/jov.23.2.10)
Supplement: Supplement 1 [file jovi-23-2-10_s001.pdf]

Supplementary Material

S1: Experiment One (Free Viewing) Presentation Mode Analysis

The linear mixed effects model included fixed effects of set size and presentation mode, as well as the interaction between set size and presentation mode. We included participant ID as a random effect [1]. Effects in the sequential presentation mode were collapsed across serial positions.

For imprecision (Figure S1A), a significant effect of set size was observed; $F(3, 18) = 43.71$, $p < 0.001$. The effect of presentation mode [$F(1, 6) = 0.43$, $p = 0.536$] and the interaction between presentation mode and set size [$F(3, 18) = 1.71$, $p = 0.201$] were not significant. Bonferroni-Holm corrected pairwise comparisons between set sizes revealed a significant difference between set size one ($M = 24.86$, $SD = 10.6$) and set size two ($M = 46.89$, $SD = 12.12$); $p < 0.001$. No other comparisons were significant; $p \geq 0.079$.

For the probability of reporting the target location (Figure 7B), a significant effect of set size was found; $F(3, 18) = 6.11$, $p = 0.005$. The effect of presentation mode [$F(1, 6) = 0.66$, $p = 0.447$] and the interaction between set size and presentation mode [$F(3, 18) = 0.83$, $p = 0.494$] were not significant. Bonferroni-Holm corrected pairwise comparisons between set sizes revealed no significant differences between set sizes; $p \geq 0.066$.

For the probability of misbinding (Figure 7C), a significant effect of set size was found; $F(3, 18) = 6.36$, $p = 0.004$. The effect of presentation mode [$F(1, 6) = 0.04$, $p = 0.849$] and the interaction between set size and presentation mode [$F(3, 18) = 0.05$, $p = 0.986$] were not significant. Bonferroni-Holm corrected pairwise comparisons between set sizes revealed no significant differences between adjacent set sizes; $p \geq 0.067$.

For the probability of guessing (Figure 7D), no significant effects of set size [$F(3, 18) = 1.61$, $p = 0.222$], presentation mode [$F(1, 6) = 1.54$, $p = 0.261$], or the interaction between set size and presentation mode [$F(3, 18) = 1.09$, $p = 0.380$] were found.

---

[1] The formula for the model was as follows for imprecision: imprecision ~ set size + presentation mode + set size*presentation mode + (1|ID)

S2: Experiment Two (Fixed Viewing) Presentation Mode Analysis

For imprecision (Figure 8A), a significant effect of set size was observed; $F(3, 24) = 10.24$, $p < 0.001$. The effect of presentation mode [$F(1, 8) = 0.64$, $p = 0.448$] and the interaction between presentation mode and set size [$F(3, 24) = 2.95$, $p = 0.053$] were not significant. Bonferroni-Holm corrected pairwise comparisons between set sizes revealed no significant differences between adjacent set sizes; $p \geq 0.123$.

For the probability of reporting the target location (Figure 8B), a significant effect of set size was found; $F(3, 24) = 12.26$, $p < 0.001$. The effect of presentation mode [$F(1, 8) = 3.86$, $p = 0.085$] and the interaction between set size and presentation mode [$F(3, 24) = 0.97$, $p = 0.422$] were not significant. Bonferroni-Holm corrected pairwise comparisons between set sizes revealed a significant difference between set size three ($M = 0.98$, $SD = 0.02$) and set size four ($M = 0.89$, $SD = 0.09$); $p < 0.001$. No other differences were significant; $p \geq 1.000$.

For the probability of misbinding (Figure 8C), a significant effect of set size was found; $F(3, 24) = 7.94$, $p < 0.001$. The effect of presentation mode [$F(1, 8) = 0.16$, $p = 0.698$] and the interaction between set size and presentation mode [$F(3, 24) = 0.09$, $p = 0.963$] were not significant. Bonferroni-Holm corrected pairwise comparisons between set sizes revealed a significant difference between set size three [$M = 0.01$, $SD = 0.02$] and set size four [$M = 0.06$, $SD = 0.06$]; $p = 0.005$. No other differences were significant; $p \geq 1.000$.

For the probability of guessing (Figure 8D), significant effects of set size [$F(3, 24) = 4$, $p = 0.019$] and presentation mode [$F(1, 8) = 12.37$, $p = 0.008$] were observed, such that guessing was significantly higher on sequential ($M = 0.04$, $SD = 0.05$) compared to simultaneous ($M = 0$, $SD = 0.01$) presented arrays. The interaction between set size and presentation mode was not significant; $F(3, 24) = 2.27$, $p = 0.106$. Bonferroni-Holm corrected pairwise comparisons between set sizes revealed a significant difference between set size three ($M = 0.01$, $SD = 0.02$) and set size four ($M = 0.05$, $SD = 0.02$); $p = 0.047$. No other differences were significant; $p \geq 1.000$.

S3: Comparison between Experiment One and Experiment Two

We examined whether the instruction to maintain central fixation affected imprecision in VSWM using linear mixed effects model (Figure 9). Set size, presentation mode and viewing condition were included as fixed effects, and participant ID was included as a random effect[2].

A significant interaction between experiment and set size was observed; $F(3, 42) = 8.64$, $p < 0.001$. Bonferroni-Holm corrected comparisons between free and fixed viewing at each set size revealed a significant difference between free viewing ($M = 24.86$, $SD = 10.6$) and fixed viewing ($M = 44.97$, $SD = 8.64$) at set size one; $p = 0.001$. No other differences were significant; $p \geq 0.467$.

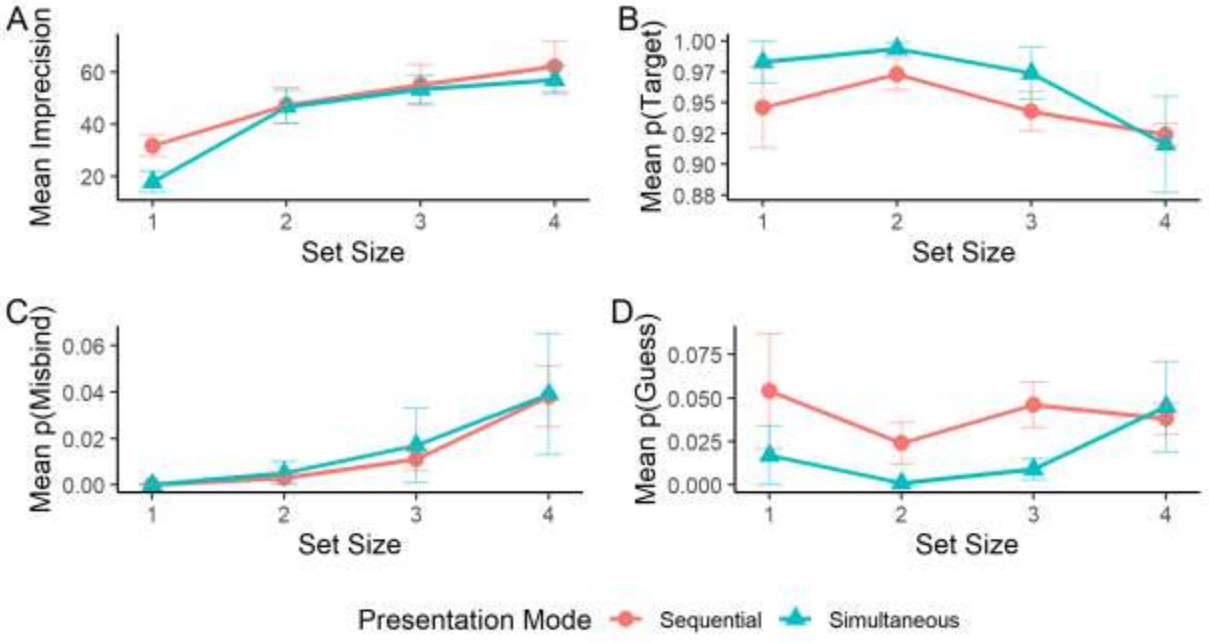

Figure 7: Mean imprecision (A), probability of reporting the target location (B), probability of misbinding (C), and probability of guessing (D) for each presentation mode as a function of set size. Effects in sequential presentation mode are collapsed across serial order. Error bars represent SEM.

[2] The formula for the model was as follows: imprecision ~ set size + presentation mode + viewing condition + set size*presentation mode + set size*viewing condition + set size*presentation mode*viewing condition + (1|ID)

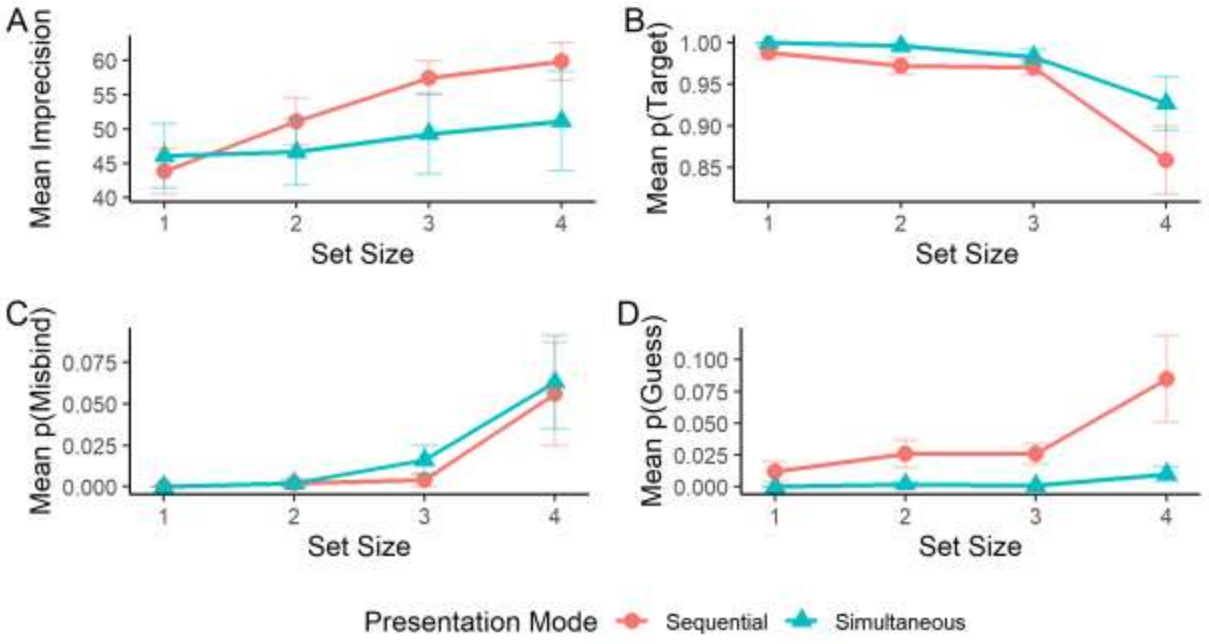

Figure 8: Mean imprecision (A), probability of reporting the target location (B), probability of misbinding (C), and probability of guessing (D) for each presentation mode as a function of set size. Effects in sequential presentation mode are collapsed across serial order. Error bars represent SEM.

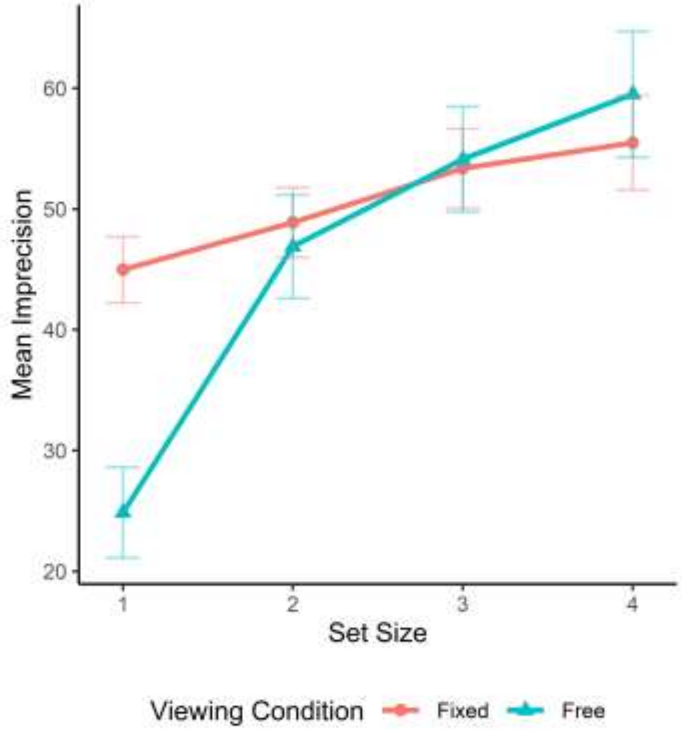

Figure 9: Mean imprecision as a function of set size in fixed viewing and free viewing. Error bars represent SEM.
